# Pharmacist-led redesign of chemotherapy delivery and supportive care: effects on infusion capacity, aseptic workload, costs and resource stewardship in oncology practice

**DOI:** 10.3389/fphar.2026.1908515

**Published:** 2026-10-28

**Authors:** Boris Dudík, Bela Mriňáková, Karolína Kimliková, Iveta Waczulíková, Orijana Krajčík, Zuzana Kiliánová, Natália Blažová, Stanislava Kosírová, Vanda Ušáková, Klaudia Gočárová, Ján Klimas

**Affiliations:** 1 Hospital Pharmacy of St. Elisabeth, St. Elisabeth Cancer Institute, Bratislava, Slovakia; 2 Department of Pharmacology and Toxicology, Faculty of Pharmacy, Comenius University Bratislava, Bratislava, Slovakia; 3 1st Department of Oncology, Faculty of Medicine, Comenius University Bratislava and St. Elisabeth Cancer Institute, Bratislava, Slovakia; 4 Department of Nuclear Physics and Biophysics, Faculty of Mathematics, Physics and Informatics, Comenius University Bratislava, Bratislava, Slovakia

**Keywords:** chemotherapy delivery, healthcare sustainability, oncology pharmacy, pharmacist-led redesign, resource stewardship, supportive care

## Abstract

**Introduction:**

Medication-related processes in oncology are complex and resource intensive, creating opportunities for treatment delays, preparation burden, medication errors, and inefficient resource utilization. Pharmacist-led interventions have been associated with improvements in medication safety, efficiency, and costs, yet evidence evaluating their system-wide impact remains limited. This study evaluated an integrated pharmacist-led, multidisciplinary redesign of medication-use processes encompassing supportive medications, infusion administration, and drug preparation in a tertiary oncology center.

**Materials and methods:**

We performed a retrospective analysis of routinely collected administration data from January to June 2023. Baseline practice (3,750 administrations) was compared with a modelled full-implementation scenario. Scheduled administration time (SAT) was used as an operational measure of infusion-unit resource use. Aseptic workload, direct acquisition costs, and assessed single-use consumable waste were estimated using prespecified model rules, measured unit weights, and 2023 institutional acquisition prices. A pragmatic February 2026 substudy compared current SAT with real administration time, reconstructed baseline equivalent SAT, and modelled full-implementation SAT.

**Results:**

Baseline cumulative SAT was 8,945 h. IV supportive medications accounted for 1,801 SAT h and 680 h of aseptic preparation time. In the modelled full-implementation scenario, total SAT decreased by 43% (−3,851 h), aseptic workload by 666.8 h, direct acquisition costs by €20,276, and assessed single-use consumable waste by 331.6 kg. In the February 2026 substudy, current SAT closely approximated real administration time and indicated that a substantial proportion of the modelled SAT reduction had already been achieved in routine practice.

**Discussion:**

To our knowledge, this is the first study to simultaneously quantify infusion service capacity, aseptic workload, direct costs, and consumable waste within a pharmacist-led, multidisciplinary oncology redesign framework. By streamlining medication-use processes across multiple stages of care, such interventions may contribute to safer medication delivery, improved resource stewardship, and greater infusion service capacity. This model represents a scalable and policy-relevant approach to supporting high-quality cancer care while promoting long-term healthcare sustainability.

## 1 Introduction

The delivery of systemic anticancer therapy is increasingly challenged by limited infusion-unit capacity and workforce constraints as patient volumes rise, treatment complexity increases, and supportive care requirements expand. In both outpatient and inpatient oncology settings, inefficient workflows and avoidable intravenous (IV) administrations can prolong scheduled administration time (SAT), increase pharmacy and nursing workload, and reduce treatment throughput without proportional clinical benefit (Huang et al., 2018; Trapani et al., 2024). At scale, these inefficiencies may affect access, waiting times, and service capacity.

Supportive medications administered with chemotherapy are an important, but often underrecognized, contributor to this burden. Antiemetics, corticosteroids, antihistamines, gastroprotective agents, and other adjunctive medicines are frequently given routinely, often by the IV route, and may reflect historical practice rather than current evidence. Although international guidelines provide clear recommendations on drug selection, dose, and route for the prevention of chemotherapy induced nausea and vomiting and infusion related reactions, real-world practice remains heterogeneous and departures from guideline are common (Barroso et al., 2024; Herrstedt et al., 2024; Kennedy et al., 2024). When repeated across many treatment visits, even small avoidable delays or unnecessary administration steps can have a meaningful impact on staff time, chair capacity, and material use.

IV chemotherapy delivery and supportive care also generate single-use consumable waste, including infusion bags, administration sets, syringes, and needles. Reducing unnecessary IV interventions may therefore support both operational efficiency and more sustainable material use. Although formal life-cycle carbon assessments are not routinely feasible at the institutional level, pragmatic service-level measures, such as mapped consumable use and waste by weight, may still provide useful information for stewardship, procurement, and quality improvement (Martin et al., 2025; Schluep et al., 2024).

Pharmacists involved in chemotherapy validation, aseptic preparation, and supportive care optimization are well placed to identify low value routine practices, translate guideline recommendations into care pathways, and support clinical governance. Pharmacist-led oncology interventions have been associated with improvements in medication safety, efficiency, and costs (Fentie et al., 2024; Han et al., 2016; Mull et al., 2024). Related literature has also examined infusion scheduling, waiting times, patient time burden, and sustainability (Fisher et al., 2018; Doshi et al., 2025; Gupta et al., 2022; Huang et al., 2018; Martin et al., 2025; Schluep et al., 2024; Sugalski et al., 2019). However, most reports of reduced infusion waiting or cycle time have arisen from multidisciplinary workflow redesign or advance preparation strategies, with interventions typically implemented through shared operational leadership rather than being coordinated and led by a clinical pharmacist (Blackmer et al., 2021; Parkes et al., 2019). Although oncology pharmacists are increasingly recognized as key contributors to treatment optimization and medication safety, pharmacist-led reports linking service redesign to reduced infusion times remain sparse and have largely focused on drug-specific protocols such as rapid rituximab conversion (Moore et al., 2021; Swan et al., 2014). Consequently, evidence is limited regarding the ability of a pharmacist to serve as the coordinating element of a multidisciplinary redesign program integrating supportive medication use, infusion administration processes, and pharmacy preparation workflows. We therefore evaluated a novel integrated pharmacist-led redesign package in a tertiary oncology center.

## 2 Materials and methods

### 2.1 Study design and setting

We conducted a retrospective model-based analysis using routinely collected administration data from a tertiary oncology center. Baseline practice comprised all systemic anticancer therapy administrations delivered from January to June 2023, before gradual implementation of standardization measures initiated in July 2023. Baseline was compared with a modelled full-implementation scenario assuming complete adoption of a pharmacist-led redesign package spanning preparation, administration logistics, and supportive medications administered with chemotherapy. The study consisted of two linked components: first, a deterministic model comparing baseline practice with modelled full implementation; and second, a pragmatic February 2026 substudy evaluating SAT against observed real administration time (RAT) and estimating implementation uptake in routine practice. The main methodological principles are described below, with detailed model rules, unit inputs, consumable counting rules, and supplementary statistical methods provided in the Supplementary Material. A schematic overview of the study design and time metrics is provided in Figure 1.

**FIGURE 1:**
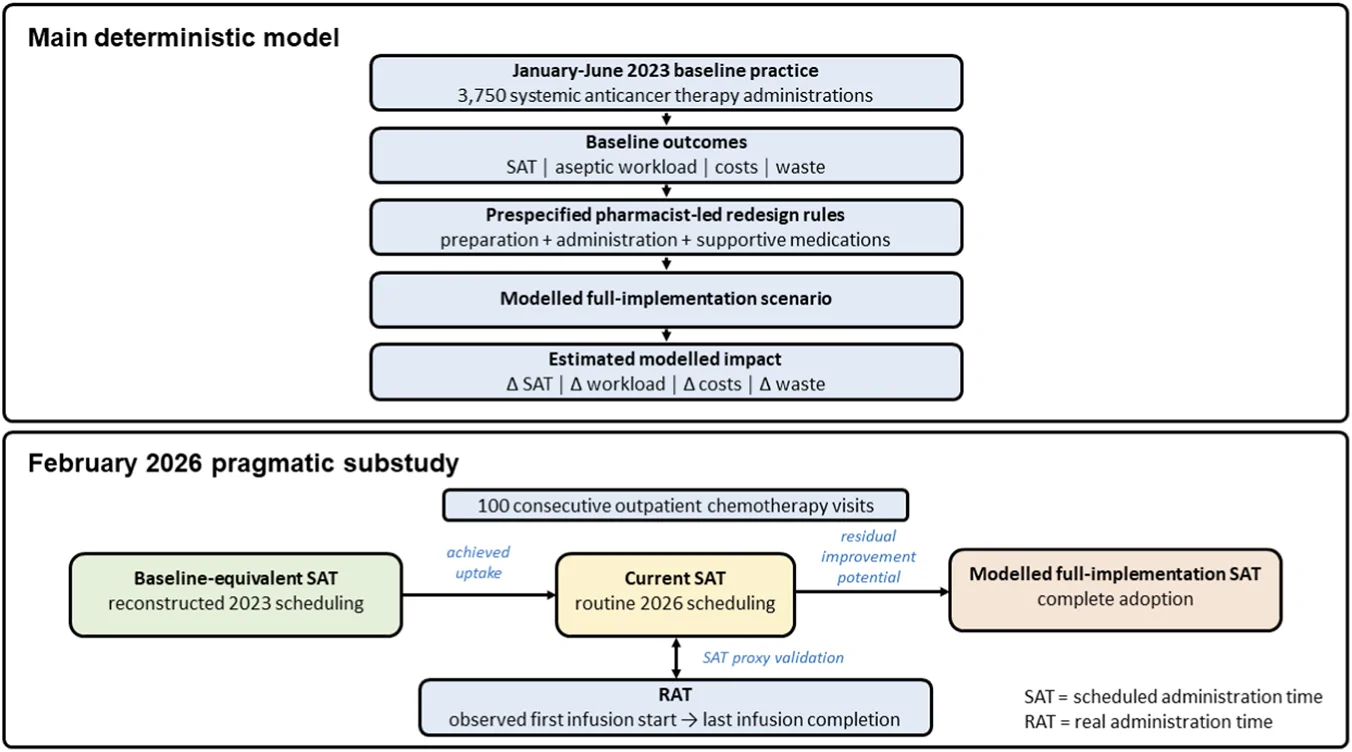
Study design and interpretation of SAT/RAT metrics. The schematic summarizes two linked components of the study. First, the main deterministic model compared January–June 2023 baseline practice with a modelled full-implementation scenario after application of prespecified pharmacist-led redesign rules spanning preparation standardization, administration optimization, and supportive medication standardization. The model quantified expected effects on scheduled administration time, aseptic workload, direct acquisition costs, and assessed single-use consumable waste. Second, the February 2026 substudy evaluated the same 100 outpatient chemotherapy visits using four time metrics: RAT, current SAT, baseline-equivalent SAT, and modelled full-implementation SAT. RAT was the observed interval from first infusion start to last infusion completion. Current SAT was compared with RAT to assess SAT as an operational proxy. Baseline-equivalent SAT was compared with current SAT to estimate achieved uptake, and current SAT was compared with modelled full-implementation SAT to estimate residual improvement potential. SAT, scheduled administration time; RAT, real administration time; IV, intravenous.

### 2.2 Study components and model structure

#### 2.2.1 Baseline chemotherapy administration and SAT construction

Administrations were extracted from the institutional chemotherapy prescribing and preparation system and included 3,750 administrations from outpatient and inpatient care. Extracted variables included regimen identity, route, scheduled infusion durations, infusion volumes/containers, and IV supportive medications administered within the same visit or admission episode. Baseline SAT was constructed from scheduled infusion durations recorded in the institutional system and included anticancer therapies, supportive infusions, and IV supportive medications administered during the chemotherapy visit or admission episode. Sequential infusions were counted as the sum of scheduled durations, whereas concurrent compatible infusions were counted by overlap, using the maximum concurrent duration. Continuous ambulatory 5-fluorouracil infusion duration beyond the treatment visit was excluded from SAT. Detailed SAT calculation rules and worked examples are provided in Supplementary Table S1A and Supplementary Appendix A.

#### 2.2.2 Modelled pharmacist-led full implementation

The modelled full-implementation scenario applied prespecified deterministic rules representing complete adoption of the pharmacist-led redesign package. The redesign package included three domains. Preparation standardization comprised use of manufacturer-recommended dilution volumes, consolidation into a single infusion container where appropriate, and smaller-volume elastomeric devices. Administration optimization comprised alignment of scheduled infusion durations with product information and local standards, shortened infusion protocols for selected agents where supported by evidence, and concurrent Y-site administration when compatibility could be verified. Supportive medication standardization comprised optimization of drug selection, dose, and route, including IV-to-oral conversion where clinically appropriate, avoidance of duplicate corticosteroids, omission of routine IV gastroprotection without clear indication, preference for non-sedating oral antihistamines where feasible, and removal of routine low-value IV adjunctive agents from default pathways while preserving individualized use when clinically indicated. Detailed intervention rules and unit inputs are provided in Supplementary Tables S1–S5 and Supplementary Appendix A.

#### 2.2.3 Pragmatic SAT validation and implementation uptake substudy

To pragmatically assess SAT as a schedule-based proxy and to provide an early uptake signal, we conducted a time-recording substudy in February 2026. In 100 consecutive outpatient chemotherapy visits observed in routine practice, a single infusion nurse recorded the time of first infusion start and the time of last infusion completion. RAT was defined as this observed interval between first infusion start and last infusion completion. Current SAT was defined as the scheduled administration time for the same February 2026 visits according to routine scheduling practice at the time of the substudy. Baseline-equivalent SAT was defined as a within-visit reconstruction of how these same February 2026 visits would have been scheduled under January–June 2023 baseline practice, using the same SAT counting rules. At our institution, chemotherapy regimens are implemented in the prescribing and preparation system as standardized regimen templates rather than patient specific free text schedules. Therefore, for each February 2026 administration, the corresponding regimen was matched to its January–June 2023 template, and the historical scheduled infusion durations, sequencing, and routine supportive medication defaults embedded in that template were reapplied to reconstruct the baseline-equivalent schedule. Modelled full-implementation SAT was defined as the scheduled administration time that would be expected for these same visits under complete adoption of the redesign package. Comparing current SAT with RAT assessed whether SAT reasonably reflected observed administration time. Comparing current SAT with baseline-equivalent SAT estimated the SAT reduction already achieved in routine practice, whereas comparing current SAT with modelled full-implementation SAT estimated residual improvement potential. Further details on reconstruction of baseline-equivalent SAT, potential sources of reconstruction bias, and substudy statistical methods are provided in the Supplementary Methods.

### 2.3 Outcome definitions and model inputs

The main modelled outcomes were total SAT, IV supportive medication-related aseptic preparation workload, direct acquisition costs, and assessed single-use consumable waste. SAT was used as a schedule-based measure of parenteral administration time and infusion-unit resource use, according to the rules summarized in Supplementary Table S1A. SAT did not capture total patient time, total chair time, pharmacy turnaround time, flushing, observation, or other workflow steps unless these were explicitly included in the institutional schedule. Oral medications were assigned 0 min of SAT because they did not require aseptic preparation or scheduled parenteral administration; SAT therefore did not include pharmacologic lead-in time or patient waiting time after oral administration. A worked example illustrating application of the model rules in baseline practice and in the modelled full-implementation scenario is shown in Figure 2 (FOLFOX).

**FIGURE 2:**
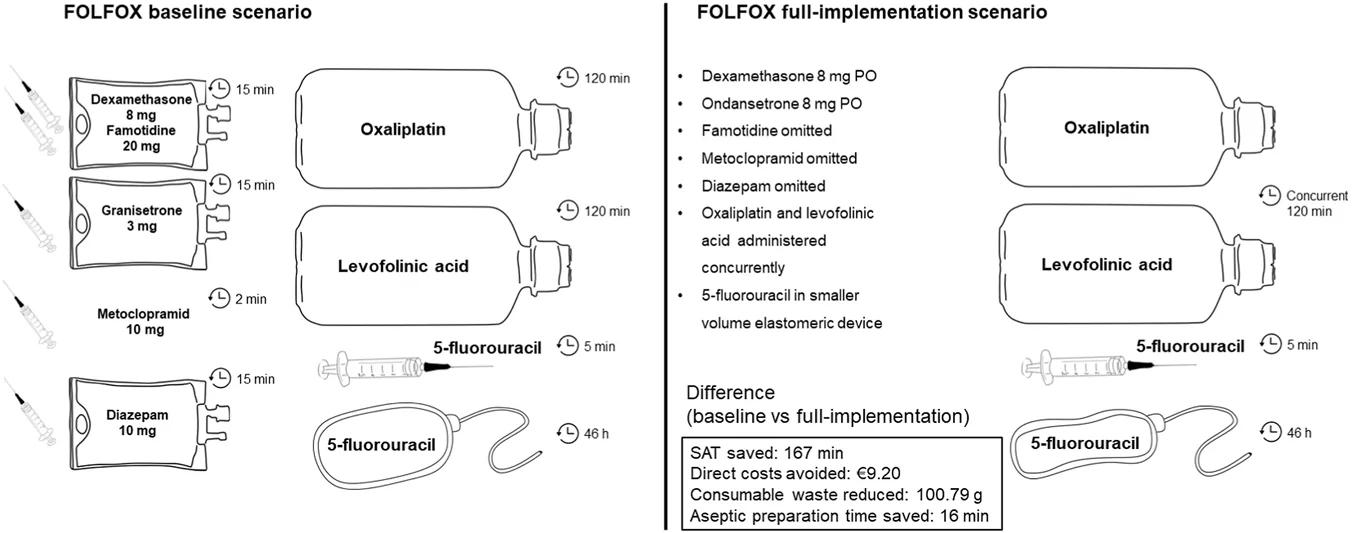
Worked example (FOLFOX) illustrating application of the model rules in baseline practice and in the full-implementation scenario. The scenario includes IV to oral conversion and omission of selected routine IV supportive medications, and concurrent administration of levofolinic acid with oxaliplatin (Y-site; overlap counted as the maximum concurrent duration). SAT was derived from scheduled infusion durations; the 46-h 5-fluorouracil infuser duration was excluded from SAT. Cost, aseptic preparation workload, and assessed single-use consumable waste outcomes refer to IV supportive medications and directly associated consumables, including one shared non-cytotoxic infusion set/line, as defined in Supplementary Tables S1–S5 and Supplementary Appendix A. FOLFOX, levofolinic acid, 5-fluorouracil and oxaliplatin; IV, intravenous; PO, oral; SAT, scheduled administration time.

Aseptic preparation workload was estimated using prespecified time-per-step inputs (Supplementary Table S1B), and costs were estimated from the institutional perspective using 2023 acquisition prices (Supplementary Table S4). For workload, cost, and waste analyses, supportive medications were defined as adjunct medications administered as part of the chemotherapy visit. Supportive infusions such as hydration, electrolyte infusions, and levofolinic acid were included in SAT where scheduled, but excluded from supportive medication-specific workload, cost, and waste modelling unless directly affected by a preparation-related container or device change.

Waste was assessed as the weight of discarded single-use consumables because this outcome was directly measurable, locally auditable, and attributable to specific medication-use changes. The analysis was limited to predefined assessed streams and did not include discarded medications, total consumable counts, or carbon footprint, which would require separate residual drug-waste, inventory, and life-cycle assessment methods. Consumable mapping, counting rules, and unit weights are provided in Supplementary Tables S2 and S3.

### 2.4 Statistical analysis

Primary counterfactual model outputs were summarized descriptively. For the February 2026 pragmatic substudy, data were summarized using descriptive and inferential statistics. Paired comparisons of current SAT versus RAT, current SAT versus baseline-equivalent SAT, and current SAT versus modelled full-implementation SAT were evaluated using paired t-tests. Agreement between current SAT and RAT was additionally assessed using Bland–Altman analysis, in which the within-visit difference was plotted against the within-visit mean. Detailed statistical methods are provided in the Supplementary Methods.

### 2.5 Ethics

The main analysis used anonymized retrospective data; ethics approval was obtained (10-2024/EK OÚSA; 11 July 2024), and the requirement for informed consent was waived because the analysis was retrospective and used anonymized routinely collected data. The February 2026 time-recording substudy consisted of operational time measurements collected during routine care for service-evaluation purposes, did not alter treatment delivery, and was analyzed in anonymized form. All research involving human data was conducted in accordance with the principles of the Declaration of Helsinki.

## 3 Results

### 3.1 Baseline chemotherapy delivery (January - June 2023)

Across 3,750 administrations, activity was dominated by solid tumor regimens (gastrointestinal, breast, gynecologic, and head and neck cancers), with FOLFOX (levofolinic acid, 5-fluorouracil and oxaliplatin), FOLFIRI (levofolinic acid, 5-fluorouracil and irinotecan), weekly and every-3-week paclitaxel, and cisplatin-based combinations being the most frequently administered regimens. Baseline cumulative SAT was 8,945 h, including anticancer therapies, supportive infusions, and intravenous supportive medications administered during the same treatment visit or admission episode. IV supportive medications accounted for 1,801 SAT hours and 680 h of aseptic preparation time. Baseline IV supportive medication costs were €30,163 (drugs €19,614; consumables €10,549), and IV supportive medication related single use consumables weighed 287.5 kg.

### 3.2 Modelled impact of pharmacist-led implementation

Under the modelled full-implementation scenario, total SAT was estimated to decrease by 43% (−3,851 h). Direct acquisition costs were estimated to decrease by €20,276, IV supportive medication related aseptic preparation workload by 666.8 h, and assessed single use consumable waste within predefined assessed streams by 331.6 kg (Table 1). These findings show simultaneous effects on service capacity, pharmacy workload, direct costs, and resource stewardship. The estimated waste reduction exceeded baseline IV supportive medication related waste alone because the assessed waste outcome also included directly attributable incremental container/device related reductions from selected preparation related measures for anticancer products.

**TABLE 1:** Summary of redesign measures and modelled impact.

Area	Measure (full-implementation change)	Potential patient and practice implications	SAT saved (h)	Cost avoided (€)	Prep time saved (h)	Waste reduced (kg)
Preparation	Dilution volumes → manufacturer recommended (e.g., pembrolizumab, bevacizumab, docetaxel)	Maintained stability	—	113	—	9.8
	Single infusion container instead of multiple (e.g., cisplatin, cetuximab, dacarbazine)	Reduced manipulation/error risk	—	2,227	Not estimated	23.9
	Smaller volume elastomeric devices	Reduced manipulation/error risk	—	530	Not estimated	12.6
Administration	Avoidance of unnecessarily prolonged infusions (align with product information, e.g., bevacizumab, cetuximab, etoposide)	Less time toxicity	415.0	—	—	—
	Shortened infusions (cisplatin, weekly paclitaxel)	Less time toxicity	722.5	—	—	—
	Concurrent infusion via Y-site: levofolinic acid + oxaliplatin/irinotecan	Less time toxicity	923.5	—	—	—
Supportive medications	Dexamethasone standardization (avoid/↓dose/IV→PO where appropriate)	Lower steroid burden; fewer IV interventions	817.8	3,061	163.6	180.3
	Hydrocortisone omitted when dexamethasone present	Avoidance of duplicate steroids	7.1	368	14.3	1.7
	5-HT3 antagonists: IV→PO; ondansetron preferred (palonosetron unchanged)	Reduced unnecessary IV exposure	382.3	5,329	76.5	31.1
	H1 antihistamines: IV bisulepine → PO levocetirizine	Reduced sedation/anticholinergic burden	124.8	1,163	31.4	9.8
	Remove routine IV H2RA/PPI	Avoidance of low-value routine IV use	56.0	5,350	271.1	26.0
	Omit routine IV metoclopramide/thiethylperazine/diazepam (individualized use outside routine pathways)	Reduced sedation and low-value IV use	401.8	2,135	110.0	36.4
Total	​	—	3,851	20,276	666.8	331.6

Abbreviations: 5-HT3, 5-hydroxytryptamine-3; H1, histamine-1; H2RA, histamine-2 receptor antagonist; IV, intravenous; PO, oral; PPI, proton pump inhibitor; SAT, scheduled administration time.

Values are modelled estimates. Waste reduction reflects predefined assessed streams and does not represent total chemotherapy related waste.

Estimated SAT reductions were driven primarily by administration standardization and reduced use of routine IV supportive medications (Table 1). Administration measures included aligning scheduled infusion durations with product information (−415 SAT hours), shortened infusion protocols for weekly paclitaxel and cisplatin (−722.5 h), and Y-site administration of levofolinic acid concurrently with oxaliplatin or irinotecan where compatible (−923.5 h). Preparation standardization contributed smaller reductions in assessed materials and costs through manufacturer recommended dilution volumes, container consolidation where appropriate, and smaller-volume elastomeric devices (Table 1). Supportive medication standardization accounted for most reductions in IV supportive medication workload, costs, and assessed waste.

### 3.3 Pragmatic validation of SAT and uptake signal (February 2026 substudy)

In a time recording substudy of 100 consecutive chemotherapy visits observed in February 2026, RAT was closely aligned with current SAT, with a mean difference (SAT – RAT) of 2.45 min (95% CI, –0.15 to 5.05; p = 0.064), supporting SAT as a practical operational proxy (Supplementary Figure S1).

Current SAT was significantly lower than reconstructed baseline equivalent SAT based on January - June 2023 practice assumptions, consistent with partial/ongoing uptake of the standardization package in routine care (95.5 vs. 143.5 min; mean difference −48.0 min, 95% CI, −58.4 to −37.6; p < 0.001). Comparison of current SAT with modelled full-implementation SAT for these visits indicated remaining improvement potential (95.5 vs. 80.2 min; mean difference 15.3 min, 95% CI, 13.2 to 17.3; p < 0.001). Taken together, these comparisons indicated that current practice had achieved approximately 76% of the total modelled SAT reduction for these visits, with residual improvement potential remaining. This substudy therefore supported both the operational validity of SAT and the real-world implementation relevance of the modelled package, while indicating substantial but incomplete implementation.

## 4 Discussion

This study provides an integrated evaluation of a pharmacist-led redesign package spanning supportive medications, administration logistics, and preparation processes. Within one framework, we quantified effects on infusion service capacity, aseptic workload, direct costs, and single use consumable waste - domains often reported separately despite being implemented together in practice. Using routinely collected high volume administration data and prespecified rules, we estimated the potential impact of full adoption rather than post-implementation effectiveness. The February 2026 substudy supported SAT as a practical operational proxy and suggested that routine practice had already captured a substantial proportion of the modelled SAT reduction. Overall, integrated redesign of infusion logistics and supportive care pathways may reduce SAT, IV supportive medication-related aseptic workload, direct costs, and assessed single-use consumable waste.

### 4.1 Operational implications for infusion services

Reducing SAT is operationally important because it serves as a schedule-based proxy for infusion-chair occupancy and infusion related workload. This remains an underreported outcome domain in pharmacist-led oncology redesign. Beyond throughput, shorter planned infusion visits may reduce patient time burden, or ‘time toxicity’, an increasingly recognized dimension of cancer treatment burden (Doshi et al., 2025; Gupta et al., 2022). This broader treatment burden is also consistent with regional evidence that cancer care can adversely affect health-related quality of life and patient’s ability to work, as shown in a study of ovarian cancer patients (Bielik et al., 2020). Patient preference for shorter treatment visits has been reported, and planned infusion times vary substantially across centers for similar regimens, suggesting a meaningful opportunity for workflow standardization (Fisher et al., 2018; Sugalski et al., 2019). SAT should be interpreted as scheduled parenteral administration time, not total chair time. At our institution, systemic anticancer therapies are not prepared in advance; preparation begins only after clinical assessment and treatment confirmation. Oral premedications are usually taken before assessment or, if not taken beforehand, administered during clinical assessment, so their lead-in time commonly overlaps with clinical assessment and/or pharmacy preparation. Therefore, IV-to-oral conversion reduces aseptic preparation and scheduled IV administration time, but may not reduce total chair time minute-for-minute, particularly if pharmacy turnaround or another workflow step is the main bottleneck.

In our modelled full-implementation scenario, SAT reductions were driven mainly by standardization of infusion durations and sequencing. Selected preparation changes also used manufacturer recommended dilution volumes and container configurations to support stability while reducing avoidable handling steps. Aligning administration schedules with product information and local standards can reduce unnecessary prolongation without changing the content of anticancer treatment. For commonly used regimens such as FOLFOX and FOLFIRI, concurrent Y-site administration of levofolinic acid with oxaliplatin or irinotecan is a practical strategy to reduce SAT while maintaining anticancer dosing (Fresenius Kabi Canada Ltd, 2023; Walker et al., 2005). Evidence also supports shorter infusion durations for selected agents after tolerance has been established, including weekly paclitaxel and cisplatin (Albuquerque et al., 2025; Dutch Society for Medical Oncology, 2024; Seidman et al., 1998). Accelerated infusions have also been described for some monoclonal antibodies, although these were outside the scope of the present redesign package during the study period (Gao et al., 2021; Hashimoto et al., 2019; Mir et al., 2012; Reidy et al., 2007; van Rijssen et al., 2025). From a service-delivery perspective, standardized default infusion durations and sequencing may improve workflow predictability, reduce avoidable chair occupancy, and potentially decrease nursing workload associated with unnecessarily prolonged infusions.

### 4.2 Supportive medications as a high-impact target

A substantial proportion of the modelled reductions in workload, costs, and assessed consumable waste arose from supportive medications administered with chemotherapy. This is important in routine practice because IV supportive care can account for a large share of aseptic preparation steps and IV-line manipulations, particularly when adjunct medications are administered intravenously by default rather than according to guideline indication and route feasibility. In our study, supportive medication pathways emerged as a particularly high-impact target for pharmacist-led redesign because they simultaneously influenced service efficiency, pharmacy workload, direct costs, and material use.

The purpose of redesign in this setting is guideline concordant optimization, not restriction of supportive care. In practice, this means removing low value routine IV components from default pathways while preserving individualized supportive care when clinically indicated, including rescue treatment where needed (Barroso et al., 2024; Herrstedt et al., 2024; Kennedy et al., 2024). Corticosteroids provide a clear example. Contemporary antiemetic guidance recommends reducing dexamethasone when an NK1 receptor antagonist is co-administered (Herrstedt et al., 2024), yet this is not consistently implemented in practice. In taxane-based regimens, dexamethasone dosing often remains based on historical routines, although observational evidence suggests that lower doses may be sufficient for many patients (Lansinger et al., 2021; Xiao et al., 2024). De-escalation strategies after uneventful prior cycles, particularly with weekly paclitaxel, have also been explored and remain under evaluation (de Castro Baccarin et al., 2019; Parinyanitikul et al., 2018; Zietse et al., 2025). Our model did not incorporate cycle by cycle omission or escalation/de-escalation; instead, it reflected the locally adopted package, including optimized indications and dosing, avoidance of duplicate corticosteroids such as hydrocortisone when dexamethasone was already present, and IV to oral conversion where clinically appropriate. Reducing steroid exposure is clinically relevant because it may lessen common short course adverse effects while also reducing unnecessary IV interventions.

Similarly, oral 5-HT3 receptor antagonists are appropriate for many patients, and IV administration is often unnecessary when oral intake is feasible (Herrstedt et al., 2024). Standardizing oral administration as the default route can reduce compounding steps and consumable use while maintaining guideline concordant prophylaxis. For antihistamines, switching from first generation sedating and anticholinergic agents to second generation agents may reduce sedation burden and improve post visit functioning in ambulatory care; it also supports de-implementation of routine IV antihistamine use when oral dosing is feasible (Beaucage-Charron et al., 2022; Durham et al., 2019; Malmberg et al., 2024). H2 receptor antagonists were historically included in paclitaxel premedication, but more recent evidence suggests that routine use is not required in many settings (Montero Pérez et al., 2023; Tsang et al., 2023). Routine IV acid suppression is also often driven by concern about corticosteroid associated gastrotoxicity, although available evidence does not support prophylaxis solely because of short course corticosteroid exposure in the absence of additional risk factors (Kumar et al., 2023; Narum et al., 2014). Standardization can therefore remove legacy add-ons from default pathways while preserving individualized prescribing for patients with clear indications, preferably by the oral route where feasible.

Routine prophylactic use of adjunctive agents such as metoclopramide, thiethylperazine, and diazepam may also reflect local tradition rather than current guideline recommendations. Metoclopramide warrants selective use given regulatory restrictions and its less favorable efficacy and safety profile compared with 5-HT3 receptor antagonists for routine prophylaxis (European Medicines Agency, 2014). Benzodiazepines should be individualized, for example, for anxiety or anticipatory nausea, and when needed is often feasible orally rather than as a routine IV component (Herrstedt et al., 2024). Removing these agents from default pathways while preserving individualized access represents a practical way to reduce unnecessary exposure and IV workload without constraining clinician discretion.

### 4.3 Resource stewardship and implementation relevance

From a sustainability perspective, this study quantified reductions in assessed single use consumable waste by weight within predefined streams. These reductions were driven mainly by avoidance of low value routine IV supportive medications and were complemented by directly attributable container and device changes from selected preparation measures. Although weight-based reporting is a pragmatic service level metric rather than a full life cycle assessment, it still provides locally actionable information for stewardship and procurement decisions (Campion et al., 2025; Cano et al., 2025; Martin et al., 2025; Schluep et al., 2024). The economic findings are also relevant for implementation: a substantial proportion of avoidable costs arose from supportive medications and the consumables needed for IV administration, rather than from anticancer drugs themselves. Even modest per administration savings may therefore accumulate meaningfully in high volume oncology services. These findings offer a transferable approach for oncology centers seeking to redesign supportive care pathways and infusion workflows under staffing, capacity, and resource constraints.

Implementation of an integrated redesign package requires clinical and operational governance, especially when changes affect prescribing defaults, aseptic preparation, and infusion scheduling. In this context, the February 2026 substudy is useful because it compared current scheduling, reconstructed baseline equivalent scheduling, and the modelled full-implementation scenario within the same visits. The substudy focused on time metrics because administration time can be recorded prospectively with minimal disruption, whereas aseptic preparation workload, costs, and consumable waste in this study were derived from model rules and institutional unit inputs and would require a separate, resource-intensive audit for direct measurement. This strengthens the study’s practical relevance by showing that the modelled framework was already partially reflected in routine practice. The February 2026 substudy indicates substantial but incomplete implementation of the modelled redesign. The close agreement between current SAT and RAT supports SAT as a practical proxy for observed parenteral administration time, but does not imply that the full-implementation scenario had been reached. Current SAT represented approximately 76% of the total modelled SAT reduction and remained 15.3 min above modelled full-implementation SAT, indicating residual improvement potential.

In addition to its broader international relevance, early implementation experience from this project helped inform and support the establishment of the Slovak Oncology Working Group on Clinical Pharmacy, a National Oncology Institute working group created not only to harmonize systemic anticancer therapy regimens and supportive care practices across Slovakia, but also to strengthen and more firmly embed clinical pharmacists in oncology practice nationwide (National Oncology Institute, 2026). In this context, the findings illustrate the potential contribution of clinical pharmacists to service optimization, supportive care standardization, and clinical governance in oncology.

### 4.4 Strengths and limitations

A key strength of this work is its integrated scope across preparation, administration logistics, and supportive medications, reflecting the interdependent nature of chemotherapy delivery. The model used transparent prespecified rules and locally measured unit inputs, supporting reproducibility and practical transferability. Unlike many pharmacist-led oncology interventions that focus on a single area, such as medication errors, antiemetic optimization, or costs alone (de Grégori et al., 2020; Moon et al., 2020; Quinn et al., 2022; Xiang et al., 2026), our approach combined preparation standardization, administration logistics, and de-implementation of low value routine supportive medication within one framework, with quantified implications for SAT, pharmacy aseptic workload, direct acquisition costs, and assessed single use consumable waste. The granularity of the modelling approach improves interpretability for real world implementation and service planning. The pragmatic substudy further strengthened the analysis by supporting the operational use of SAT and providing an early implementation signal.

Several limitations should be considered. First, the analysis was model based rather than a pre-post effectiveness evaluation, and the realized impact will depend on uptake and adherence. A model-based approach was nonetheless appropriate here because the primary aim was to estimate the potential service level impact of full adoption to inform planning and governance decisions, rather than to measure post implementation effectiveness; the February 2026 substudy provides a complementary early implementation signal. Second, the model assumed full adoption and did not include sensitivity or uptake scenarios; the estimates should therefore be interpreted as upper bound estimates. Third, indirect costs such as staffing, infrastructure, and overhead were not included. Fourth, waste estimation was limited to predefined assessed streams for IV supportive medications and directly attributable preparation related container and device changes; other waste streams and formal carbon footprint assessment were outside the scope of this study. Fifth, clinical outcomes such as nausea control, hypersensitivity reactions, and patient reported experience were not measured and should be evaluated prospectively. Finally, SAT is schedule based and may differ from observed chair occupancy because some workflow components are not captured, although the February 2026 substudy supported its use as a practical operational proxy.

## 5 Conclusion

To our knowledge, this is the first study to quantify infusion service capacity, aseptic preparation workload, direct medication-related costs, and single-use consumable waste simultaneously within a pharmacist-led multidisciplinary oncology redesign framework. In this modelled full-adoption scenario, the intervention reduced scheduled administration time, aseptic preparation workload, direct acquisition costs, and assessed consumable waste across domains that are typically evaluated separately. Beyond operational efficiency, the findings demonstrate how pharmacist-led redesign of medication-use processes can serve as a systems-level intervention supporting safer, more standardized, and more sustainable delivery of oncology care. The framework provides a transferable implementation model that integrates medication use standardization, resource stewardship, and multidisciplinary collaboration, while highlighting the potential of clinical pharmacists to lead complex service redesign initiatives and contribute to governance strategies addressing capacity, workforce, and sustainability challenges across diverse healthcare settings.

Future studies should evaluate the real-world impact of such redesign strategies on medication safety, implementation metrics, patient-reported outcomes, and survival endpoints.

## Data Availability

The original contributions presented in the study are included in the article/Supplementary Material, further inquiries can be directed to the corresponding authors.
