# Supplementary material for: Pharmacist-led redesign of chemotherapy delivery and supportive care: effects on infusion capacity, aseptic workload, costs and resource stewardship in oncology practice
Source: Front Pharmacol. 2026 Oct 28;17:1908515. doi: 10.3389/fphar.2026.1908515 (PMC13752116; doi:10.3389/fphar.2026.1908515)
Supplement: Supplementary file 1 [file Supplementaryfile1.docx]

**Supplementary Methods and Model Inputs**

Supplementary Tables S1–S5 summarize the prespecified rules and unit inputs used to calculate scheduled administration time (SAT), aseptic preparation workload, IV supportive medication consumable use, unit weights, unit acquisition costs, and supportive medication standardization rules applied in the modelled full-implementation scenario. Inputs were applied to baseline utilization data and to the counterfactual scenario to support reproducibility. The primary counterfactual results are model-derived estimates. The February 2026 substudy provided pragmatic operational time measurements to assess agreement between SAT and RAT and to estimate implementation uptake, but it was not designed as a full post-implementation effectiveness evaluation.

**Study period selection and implementation context**

The January – June 2023 baseline period was selected to represent routine high volume practice before gradual implementation of standardization measures initiated in July 2023, primarily in the preparation and administration domains.

**Data extraction**

All systemic anticancer therapy administrations delivered during the baseline period were extracted from the institutional chemotherapy prescribing and preparation system. Extracted variables included regimen identity, route, scheduled infusion durations, infusion volumes/containers, IV supportive medications and supportive infusions administered within the same visit/admission episode, and variables required to apply predefined consumables counting rules (Supplementary Table S2).

**Scope and outcomes**

SAT was derived from scheduled infusion durations for all parenteral therapies administered during the chemotherapy visit, including anticancer agents, supportive infusions, and IV supportive medications. Concurrent infusions (e.g., Y-site) were handled by overlap (maximum concurrent duration). Evidence supported shortened infusion protocols were modelled only for weekly paclitaxel and cisplatin.

Supportive infusions (e.g., hydration/electrolyte infusions and levofolinic acid administered with chemotherapy) were included in SAT where scheduled but excluded from supportive medication–specific workload, cost, and assessed waste modelling because they were not targeted by the redesign package. These outcomes were restricted to (i) IV supportive medications recorded in the prescribing system, including directly associated IV consumables, and (ii) directly attributable container/device changes from selected preparation measures (manufacturer recommended smaller containers, consolidation into a single container where applicable, and smaller elastomeric devices). IV supportive medications not modified by the package (e.g., palonosetron) were retained to ensure consistent accounting across baseline and counterfactual scenarios. Oral medication costs were included only where an IV to PO switch was explicitly modelled; for H1 antihistamines, the counterfactual included both IV to PO switching and a molecule change (IV bisulepine to oral levocetirizine).

**Exclusions (not modelled)**

No additional flush, observation, connection/disconnection, or other nursing workflow time was added beyond scheduled infusion durations. Continuous ambulatory 5-fluorouracil infusion duration beyond the visit was excluded from SAT. Waste streams outside the predefined consumables list were not assessed. NK1 receptor antagonist optimization was not modelled because these agents were already administered orally and considered guideline concordant at baseline.

**Redesign package (overview)**

The redesign package was developed by oncology pharmacists based on international guidelines, product characteristics and manufacturer instructions, published evidence supporting infusion time optimization and supportive medication safety, and local operational feasibility. It comprised three domains:

**Preparation standardization:** manufacturer recommended dilution volumes, consolidation into a single infusion container where appropriate, and optimization of elastomeric infusion devices to support physicochemical stability, reduce handling steps, and minimize consumable use.

**Administration optimization:** alignment with recommended infusion durations, evidence supported shortened protocols, and concurrent administration via Y-site when compatibility could be verified. Shortened infusion protocols were modelled only for weekly paclitaxel and cisplatin (Supplementary Table S1A; Supplementary Appendix A).

**Supportive medication standardization:** optimization of drug selection, dosing, and route in accordance with guideline recommendations, including avoidance of duplicate corticosteroids, preference for non-sedating antihistamines where feasible, omission of routine IV gastroprotection in the absence of clear indications, and restriction of non-evidence based adjunctive agents. Oral ondansetron and oral dexamethasone were modelled as default routes where clinically appropriate, while palonosetron remained unchanged. Agents discontinued from routine IV use (e.g., metoclopramide, thiethylperazine, diazepam) were assumed to remain available as optional PRN oral supportive medication; because PRN use is individualized, oral PRN costs were not modelled (Supplementary Table S5B).

**Core calculation inputs**

SAT calculation. Rules are summarized in Supplementary Table S1A, with worked examples in Supplementary Appendix A.

**Aseptic preparation workload.** Time per step inputs were derived from direct observation of routine aseptic compounding and consensus between two experienced oncology pharmacists. Inputs were applied identically in baseline and counterfactual scenarios; interpretation therefore focuses on relative differences. Unit inputs are provided in Supplementary Table S1B.

**Cost estimation.** Costs were estimated from the institutional perspective using 2023 institutional acquisition prices (including VAT). For IV to oral optimization, IV costs comprised the drug plus directly associated IV consumables, whereas oral costs comprised only the oral dosage form administered on the same treatment day. Indirect costs (staffing, infrastructure, overheads) were not included. Unit acquisition costs are provided in Supplementary Table S4.

**Consumable waste (assessed streams).** Waste outcomes refer to predefined assessed streams and do not represent total chemotherapy related waste. IV supportive medication related consumable waste was quantified using standardized consumables mapping/counting rules (Supplementary Table S2) and measured unit weights (Supplementary Table S3). For selected preparation standardization measures with deterministic, directly attributable changes in infusion containers/devices, incremental waste differences were estimated using measured unit weights and observed frequencies. Where bottle based cytotoxic preparation required short preparation lines/spikes, these were counted only for the specific container changes modelled to avoid comprehensive attribution of cytotoxic related consumables (Supplementary Appendix A).

**Supplementary Methods: February 2026 substudy—statistical analysis**

**Objectives and endpoints**

The February 2026 substudy had two objectives: (1) to assess agreement between SAT and observed real administration time (RAT), and (2) to provide an early uptake signal by comparing current SAT with reconstructed baseline equivalent SAT and modelled full-implementation SAT within the same visits. RAT was defined as the interval between first infusion start and last infusion completion. Baseline equivalent SAT was defined as a within visit reconstruction for the same February 2026 administrations, specified as if scheduled under baseline (January–June 2023) practice; no separate 2023 patient sample was used.

**Reconstruction of baseline-equivalent SAT (within visit counterfactual)**

For each February 2026 outpatient administration, we reconstructed how the same visit would have been scheduled under baseline practice by matching the administered regimen to the corresponding January–June 2023 regimen template in the institutional chemotherapy prescribing and preparation system. In this system, systemic anticancer therapy regimens are implemented as standardized templates/order sets rather than patient-specific free-text schedules; therefore, scheduled infusion durations, sequencing of parenteral administrations, and routine supportive/prophylactic medication defaults were embedded at regimen level. Historical baseline template settings were then reapplied to the same February 2026 visits. Components targeted by the redesign package were reassigned to baseline assumptions, whereas components not targeted by the package were kept unchanged. This approach generated a within visit counterfactual baseline-equivalent SAT for the same 100 February 2026 visits, rather than comparing the 2026 substudy with a separate 2023 patient sample.

Because baseline-equivalent SAT was reconstructed retrospectively, some misclassification is possible, particularly if regimen templates changed over time or exceptional non-standard scheduling decisions were not captured. However, reconstruction was based on standardized January–June 2023 regimen templates/order sets and the same SAT counting rules as the main model. Baseline-equivalent SAT should therefore be interpreted as a counterfactual estimate rather than directly observed historical administration time.

**Statistical methods**

For the February 2026 pragmatic substudy, the collected data were summarized using descriptive and inferential statistical methods. Paired comparisons of current SAT versus RAT, current SAT versus baseline equivalent SAT, and current SAT versus modelled full-implementation SAT were evaluated using paired t-tests. Agreement between current SAT and RAT was additionally assessed using Bland–Altman analysis, in which the within-visit difference was plotted against the within-visit mean. All tests were conducted at a significance level of α = 0.05, and two-sided p values are reported. Statistical analyses were performed using StatsDirect® 4.0.4 (StatsDirect Ltd in collaboration with The University of Liverpool, Cheshire, UK).

**Supplementary Results: February 2026 substudy**

**Agreement between RAT and SAT (Bland–Altman)**

The substudy was restricted to outpatient chemotherapy administrations. Agreement between real administration time (RAT) and scheduled administration time (SAT) was evaluated using Bland–Altman analysis (Supplementary Figure S1). The mean difference (SAT − RAT) was 2.45 minutes, indicating that SAT modestly overestimated observed administration time on average (p = 0.064). The 95% limits of agreement in the Bland–Altman analysis were −23.2 to 28.1 minutes.

**Uptake signal and residual potential (baseline-equivalent vs current vs modelled SAT)**

Within the same 100 visits, current SAT was lower than reconstructed baseline-equivalent SAT (95.5 vs 143.5 minutes; mean difference −48.0 minutes, 95% CI, −58.4 to −37.6; p < 0.001), indicating that a substantial portion of the modelled SAT reduction had already been realized in routine practice by February 2026. Current SAT remained higher than modelled full-implementation SAT (95.5 vs 80.2 minutes; mean difference 15.3 minutes, 95% CI, 13.2 to 17.3; p < 0.001), indicating residual improvement potential.

**Figure S1.** The Bland–Altman plot displays the difference between each individual pair of SAT/RAT measurements against their average. The overall mean difference (2.45 minutes; p = 0.064) is shown as a grey line, with the 95% limits of agreement ranging from −23.2 to 28.1 minutes. The line of equality corresponds to zero (not shown).

**Supplementary Table S1. Model rules and unit inputs**

**Supplementary Table S1A. Scheduled administration time (SAT): deterministic calculation rules**

SAT was defined as the total scheduled infusion time for all parenteral medications administered during the chemotherapy visit, as recorded in the institutional scheduling system.

| **Rule** | **Operational definition** |
| --- | --- |
| Sequential infusions | SAT = sum of scheduled infusion durations. |
| Concurrent infusions (Y-site) | SAT = maximum overlapping duration (overlap counted once). |
| IV-to-PO switch | Oral administrations were assigned 0 minutes of SAT. |
| Excluded from SAT | Flush times and post-infusion observation not captured in schedules; connection/disconnection time unless explicitly scheduled; continuous ambulatory 5-fluorouracil infusion duration beyond the visit. |

Abbreviations: IV, intravenous; PO, oral; SAT, scheduled administration time.

**Supplementary Table S1B. Preparation time per step inputs**

Time per step inputs were derived from direct observation of routine aseptic compounding and consensus between two experienced oncology pharmacists. Times represent average preparation time per item under routine conditions and include typical supporting steps (material gathering/transport, labeling, surface disinfection, and aseptic manipulation to obtain a ready-to-administer product). Inputs were applied identically in baseline and full-implementation scenarios; interpretation therefore focuses on relative differences.

| **Preparation step (aggregated)** | **Applies to** | **Time per step (min)** |
| --- | --- | --- |
| IV push (bolus) – no reconstitution | IV push without reconstitution | 2 |
| IV push (bolus) – with reconstitution | IV push requiring reconstitution | 4 |
| Short IV infusion (bag) – no reconstitution | Short IV infusion prepared in an infusion bag without reconstitution | 3 |
| Short IV infusion (bag) – with reconstitution | Short IV infusion prepared in an infusion bag requiring reconstitution | 5 |

**Notes:** Time unit is per prepared item. Source: direct observation and pharmacist consensus.

**Abbreviations:** IV, intravenous.

**Supplementary Table S2. Consumables mapping and counting rules**

Consumable use was mapped deterministically based on local standard operating procedures and applied identically to baseline utilization data and the counterfactual full-implementation scenario. Two streams were distinguished: (i) IV supportive medication consumables (fully mapped) and (ii) incremental container/device related changes attributable to selected preparation standardization measures for anticancer products. The incremental approach quantified directly attributable differences and was not intended as a full inventory of cytotoxic preparation waste.

**IV supportive medications (fully mapped).** At our institution, IV supportive medications are prepared in infusion bags in the aseptic unit and categorized as (i) IV push (bolus) or (ii) short IV infusion in a bag. For IV push administrations, one syringe (3 mL or 5 mL, depending on drug volume) and one needle were counted per administration. For short IV infusions, consumables included one syringe, one needle, and one infusion bag (typically 100 mL or 250 mL, depending on final volume).

**Shared non-cytotoxic infusion set/line.** In local practice, short IV supportive infusions are typically administered via a single shared non-cytotoxic infusion set/line whenever possible. Accordingly, one infusion set was counted per visit if ≥1 short IV supportive infusion requiring a line was administered, not per individual drug, unless separate administration was clinically required.

**Anticancer products prepared in bottles (incremental changes only).** Bottle related consumables were not inventoried across all anticancer administrations. Instead, bottle/device related materials were quantified only when directly affected by selected preparation standardization measures, as incremental differences between baseline and counterfactual:

• Bottle volume changes were quantified as the difference in unit weights between bottle sizes (larger − smaller).

• A short bottle specific preparation tubing/spike was included only for interventions that changed bottle count, because spike use scales with bottle number.

• Elastomeric device size changes were treated analogously, including directly attributable differences in device related materials and any associated change in carrier solution bottle use where applicable.

| **Category / intervention type** | **Consumables counted** | **Counting rule** | **Rationale / local practice** |
| --- | --- | --- | --- |
| Short IV supportive infusion prepared in aseptic unit (infusion bag) | Infusion bag (100 mL or 250 mL), syringe (3 mL or 5 mL), needle; shared infusion set/line | Per dose: 1 bag + 1 syringe + 1 needle. Infusion set: 1 per visit if ≥1 short IV supportive infusion requiring a line is administered ('supportive medication block'). | Supportive medications are prepared in bags and administered through a shared non-cytotoxic line whenever possible. |
| IV push (bolus) supportive medication prepared in aseptic unit | Syringe (3 mL or 5 mL), needle | Per administration: 1 syringe + 1 needle | No infusion container or infusion set required. |
| Shared non-cytotoxic infusion set/line | Non-cytotoxic infusion set/line | 1 per visit if ≥1 short IV supportive infusion requiring a line is administered; not per drug unless separate administration is clinically required | Local practice uses a single shared line for short supportive infusions. |
| Bottle volume reduction (anticancer products; incremental) | Smaller infusion bottle (weight difference only) | Waste/cost impact = larger bottle − smaller bottle, using unit weights (and unit costs if applicable) | Incremental difference between bottle sizes. |
| Bottle volume increase (anticancer products; incremental) | Larger infusion bottle (weight difference only) | Additional waste/cost = larger bottle − smaller bottle | Increased volume requires additional container material. |
| Consolidation into a single bottle (anticancer products; incremental) | Fewer infusion bottles; short bottle specific preparation tubing/spike | Δ bottles = baseline bottle count − counterfactual bottle count. Δ spikes = same difference, counted only for this bottle-count change. | Bottle count is reduced; spikes are included only where bottle count changes directly. |
| Elastomeric device size change (e.g., 230 mL → 110 mL; incremental) | Elastomeric device and any directly attributable carrier-solution bottle change | Difference in device related materials between scenarios; associated bottle differences included only when directly changed by the intervention | Only directly affected device-related materials were counted (incremental approach). |

**Supplementary Table S3. Consumable material weights and weighing procedure**

Consumables were weighed as discarded in routine practice, including packaging routinely disposed of with the item (e.g., overwrap, caps/covers, attached components). Where relevant, items were emptied and air-dried before weighing to remove residual liquids. Mean unit weights were calculated from three repeated measurements using a calibrated laboratory scale.

| **Consumable material** | **Mean weight (g)** |
| --- | --- |
| 1000 mL bottle | 46.6 |
| 500 mL bottle | 31.1 |
| 250 mL bottle | 22.7 |
| 100 mL bottle | 19.8 |
| 250 mL bag | 18.28 |
| 100 mL bag | 13.28 |
| 3 mL syringe | 3.98 |
| 5 mL syringe | 5.93 |
| Needle | 1.1 |
| Shared non-cytotoxic infusion set/line | 28.6 |
| IV line for preparation of cytotoxic drug | 15 |
| IV line for preparation of cytotoxic drug with IV filter | 24 |

**Supplementary Table S4. Unit acquisition costs**

Unit costs were taken from 2023 institutional acquisition prices recorded in the hospital purchasing system and include VAT. Oral medication costs were calculated per tablet/capsule. For IV to PO comparisons, IV costs comprised the drug plus all directly associated IV consumables, whereas PO costs comprised only the oral dosage form administered on the same treatment day. Reimbursement status was not assessed; prices are intended for within-institution comparisons.

| **Drug or consumable** | **Acquisition price (€)** |
| --- | --- |
| Bisulepine 1 mg IV | 1.08 |
| Levocetirizine 5 mg PO | 0.09 |
| Famotidine 20 mg IV | 1.34 |
| Pantoprazole 20 mg IV | 1.80 |
| Dexamethasone 8 mg IV | 0.28 |
| Dexamethasone 4 mg PO | 0.58 |
| Granisetron 3 mg IV | 5.86 |
| Granisetron 2 mg PO | 4.84 |
| Ondansetron 8 mg IV | 0.39 |
| Ondansetron 8 mg PO | 1.46 |
| Palonosetron 0.5 mg IV | 22.00 |
| Metoclopramide 10 mg IV | 0.17 |
| Thiethylperazine 6.5 mg IV | 0.50 |
| Hydrocortisone 100 mg IV | 1.51 |
| Diazepam 10 mg IV | 0.21 |
| 1000 mL bottle NS | 1.24 |
| 500 mL bottle NS | 0.94 |
| 500 mL bottle G5% | 0.85 |
| 250 mL bottle NS | 0.83 |
| 250 mL bottle G5% | 0.80 |
| 100 mL bottle NS | 0.83 |
| 100 mL bottle G5% | 0.62 |
| 250 mL bag NS | 0.83 |
| 100 mL bag NS | 0.78 |
| 3 mL syringe | 0.10 |
| 5 mL syringe | 0.14 |
| Needle | 0.04 |
| Shared non-cytotoxic infusion set/line | 0.83 |
| IV line for preparation of cytotoxic drug | 3.08 |
| IV line for preparation of cytotoxic drug with IV filter | 5.82 |

**Abbreviations:** G5%, 5% glucose; IV, intravenous; NS, normal saline; PO, oral.

**Supplementary Table S5. Counterfactual supportive medication standardization rules**

**S5A. Regimen level emetogenicity classification and routine day-1 antiemetic prophylaxis rules (counterfactual)**

Regimen emetogenicity was assigned at the regimen level (highest risk component) using the institutional regimen library aligned with the MASCC/ESMO 2023 update (Herrstedt et al., 2024) and used to define routine day-1 prophylaxis (Table S5A). The model prioritized oral administration where feasible (oral administrations assigned 0 minutes of SAT). Palonosetron was not targeted and remained unchanged (including route). No patient level risk stratification was applied; rules were regimen based.

**Table S5A. Regimen-level day-1 prophylaxis rules (counterfactual)**

| **Emetogenicity category** | **Classification approach (regimen level)** | **Counterfactual routine prophylaxis (day 1)** |
| --- | --- | --- |
| Minimal | As per regimen library (MASCC/ESMO-aligned) | No routine antiemetic prophylaxis |
| Low | As per regimen library (MASCC/ESMO-aligned) | Single-agent oral 5-HT3 RA (default: ondansetron 8 mg PO) |
| Moderate | As per regimen library (MASCC/ESMO-aligned) | Oral 5-HT3 RA (default: ondansetron) + dexamethasone (dose per regimen library) |
| High | As per regimen library (MASCC/ESMO-aligned) | Guideline-concordant prophylaxis per regimen library; dexamethasone day-1 dose adjusted when NK1 RA used |

**Abbreviations:** 5-HT3 RA, 5-hydroxytryptamine-3 receptor antagonist; IV, intravenous; NK1 RA, neurokinin-1 receptor antagonist; PO, oral; SAT, scheduled administration time.

**S5B. Cross cutting supportive medication standardization modelling rules (full-implementation scenario)**

In addition to regimen level rules in Table S5A, the counterfactual scenario applied cross-cutting standardization rules addressing recurrent baseline patterns (Table S5B), including NK1-associated dexamethasone day-1 adjustment, standardized taxane dexamethasone prophylaxis, avoidance of duplicate corticosteroids, and IV→PO defaulting where feasible (oral administrations assigned 0 minutes of SAT). Cycle to cycle omission/de-escalation strategies after uneventful administrations were not modelled.

**Table S5B. Cross-cutting rules (counterfactual)**

| **Domain / rule** | **Baseline pattern addressed** | **Counterfactual (full-implementation) rule** |
| --- | --- | --- |
| Dexamethasone dose with NK1 RA (high emetogenic risk) | Dexamethasone 20 mg co-administered with NK1 RA prophylaxis | Modelled as dexamethasone 12 mg on day 1 when an NK1 RA was co-administered (MASCC/ESMO-aligned). |
| Taxane prophylaxis (dexamethasone) | Dexamethasone 20 mg IV used routinely for taxanes | Modelled as dexamethasone 8 mg (route per IV→PO rule). Omission strategies after uneventful administrations were not modelled. |
| Low emetogenic risk regimens (antiemetic choice) | Dexamethasone used as sole routine antiemetic in some low-risk regimens | Modelled as single-agent oral 5-HT3 RA (default: ondansetron 8 mg PO) instead of dexamethasone (excluding paclitaxel, docetaxel, and cetuximabcontaining regimens). |
| Minimal emetogenic risk regimens | Routine prophylaxis occasionally used | Modelled as no routine antiemetic prophylaxis. |
| Hydrocortisone duplication | Hydrocortisone administered despite concomitant dexamethasone within the same visit | Modelled as omitted whenever dexamethasone was already administered (avoid duplicate corticosteroid exposure). |
| Route assignment (IV→PO) | IV route used where oral route was feasible | Where an IV→PO switch was feasible per institutional policy, oral administration was modelled as the default route and assigned 0 minutes of SAT. |

**Abbreviations:** 5-HT3 RA, 5-hydroxytryptamine-3 receptor antagonist; IV, intravenous; NK1 RA, neurokinin-1 receptor antagonist; PO, oral; SAT, scheduled administration time.

**Supplementary Appendix A. Worked Example of Model Calculations (revised; single example)**

This appendix provides a worked example illustrating deterministic calculations of SAT, IV supportive medication–related aseptic preparation workload, assessed single-use consumable waste (by weight), and direct acquisition costs using the model rules and unit inputs described in Supplementary Tables S1–S5. The example (FOLFOX) demonstrates supportive medication standardization (IV→PO switching and omission of selected routine IV supportive medications) and administration optimization via Y-site overlap.

**Worked Example. FOLFOX (baseline)**

SAT corresponds to scheduled infusion time recorded in the institutional scheduling system. Preparation time, consumable weight, and costs are reported for IV supportive medications (drug plus directly associated IV consumables) and the Shared non-cytotoxic infusion set/line. Anticancer and IV supportive infusions are listed for SAT context only.

| **Medication** | **SAT (min)** | **Prep time (min)** | **Waste (g)** | **Cost (€)** |
| --- | --- | --- | --- | --- |
| Dexamethasone 8 mg + famotidine 20 mg infusion (250 mL bag) | 15 | 8 | 30.39 | 2.77 |
| Granisetron 3 mg infusion (100 mL bag) | 15 | 3 | 18.36 | 6.78 |
| Metoclopramide 10 mg IV bolus | 2 | 2 | 5.08 | 0.31 |
| Diazepam 10 mg infusion (100 mL bag) | 15 | 3 | 18.36 | 1.13 |
| Levofolinic acid infusion | 120 | Not estimated | Not estimated | Not estimated |
| Oxaliplatin infusion | 120 | Not estimated | Not estimated | Not estimated |
| 5-fluorouracil bolus | 5 | Not estimated | Not estimated | Not estimated |
| 5-fluorouracil infusor (ambulatory; duration excluded) | excluded | Not estimated | Not estimated | Not estimated |
| **Shared non-cytotoxic infusion set/line** | — | — | 28.60 | 0.83 |
| **TOTAL** | **292** | **16** | **100.79** | **11.82** |

**Worked Example. FOLFOX (full-implementation scenario)**

Counterfactual values reflect the modelled scenario: dexamethasone IV→PO, famotidine omitted, 5-HT3 RA switched to oral ondansetron, metoclopramide and diazepam omitted, and levofolinic acid administered concurrently with oxaliplatin (Y-site; SAT counted by maximum overlap).

| **Medication** | **SAT (min)** | **Prep time (min)** | **Waste (g)** | **Cost (€)** |
| --- | --- | --- | --- | --- |
| Dexamethasone 8 mg (PO); famotidine omitted | 0 | 0 | 0 | 1.16 |
| Ondansetron 8 mg (PO) | 0 | 0 | 0 | 1.46 |
| Metoclopramide 10 mg | omitted | 0 | 0 | 0.00 |
| Diazepam 10 mg | omitted | 0 | 0 | 0.00 |
| Levofolinic acid + oxaliplatin (concurrent, Y-site) | 120 | Not estimated | Not estimated | Not estimated |
| 5-fluorouracil bolus | 5 | Not estimated | Not estimated | Not estimated |
| 5-fluorouracil infusor (ambulatory; duration excluded) | excluded | Not estimated | Not estimated | Not estimated |
| **TOTAL** | **125** | **0** | **0** | **2.62** |

**Difference (baseline − full-implementation scenario):** 167 min SAT; 16 min preparation time; 100.79 g assessed waste; €9.20.

**Abbreviations:** 5-HT3 RA, 5-hydroxytryptamine-3 receptor antagonist; IV, intravenous; PO, oral; SAT, scheduled administration time; Y-site, Y-site connector.
